# The Swedish moral injury symptoms scale–health professional: psychometric evaluation in a sample of hospital-based healthcare professionals across Sweden

**DOI:** 10.3389/fpsyg.2026.1923213

**Published:** 2026-09-04

**Authors:** Anna Hjalmarsson, Jan Grimell, Jelle van Gurp, Milou Looijmans, Tine Molendijk, Alexandra Gomes Almeida, Niek Kok, Andrea Feijo Mello, Bruno Messina Coimbra

**Affiliations:** 1 Department of Sociology, Umeå University, Umeå, Sweden; 2 Radboud University Medical Center, Radboud Institute for Health Sciences (IQ Health), Nijmegen, Netherlands; 3 Department of Military Management Studies, Faculty of Military Sciences, Netherlands Defense Academy, Breda, Netherlands; 4 Department of Political Science, Radboud University Nijmegen, Netherlands; 5 Department of Psychiatry, Universidade Federal de São Paulo (UNIFESP), São Paulo, Brazil; 6 Department of Ethics and Law, Leiden University Medical Center, Leiden, Netherlands; 7 Amsterdam UMC, Department of Psychiatry, Amsterdam Public Health and Global Health Research Institute, University of Amsterdam, Amsterdam, Netherlands

**Keywords:** COVID-19 pandemic, healthcare professionals, instrument validation, MISS-HP, moral injury, moral injury symptoms scale—health professional, psychometric evaluation, Sweden

## Abstract

**Background:**

The COVID-19 pandemic has increased attention to moral injury (MI) among healthcare professionals (HCPs). MI refers to the psychological distress that may arise following exposure to events that violate individuals’ deeply held moral beliefs. The Moral Injury Symptoms Scale–Health Professional (MISS-HP) is an instrument designed to assess MI symptoms among HCPs. Although the MISS-HP has been evaluated in several countries, divergent psychometric solutions have been reported, and the instrument has not yet been evaluated in Sweden.

**Objective:**

To evaluate the psychometric properties of the Swedish version of the MISS-HP among hospital-based HCPs.

**Methods:**

HCPs were recruited using an online survey distributed across Swedish hospitals. Exploratory factor analysis (EFA) was performed to investigate the factorial structure of the MISS-HP. Internal consistency was assessed using McDonald’s Omega and construct validity through measures of psychological distress. Recevier operating characteristic (ROC) analysis was used to estimate a candidate cut-off score.

**Results:**

The sample included 585 HCPs (mean age = 45.95 years, 72.2% women, 51.4% nurses). EFA supported a two-factor solution (Guilt/Shame, Trust/Meaning) comprising six of the original MISS-HP ten items and showed good fit [*χ*^2^(4) = 9.07, *p* = 0.059; RMSEA = 0.046; TLI = 0.975; RMSR = 0.018], accounting for 43.6% of the total variance. McDonald’s Omega indicated good internal consistency (*ω* = 0.80). Measures of psychological distress were moderately associated with MISS-HP scores and ROC analysis identified a candidate cut-off score of ≥ 20.

**Conclusion:**

The resulting six-item Swedish MISS-HP demonstrated satisfactory psychometric performance for measuring MI symptoms in the sample.

## 1 Introduction

Moral injury (MI) refers to the profound psychological distress that may arise following exposure to events that violate an individual’s deeply held moral beliefs and values (Litz et al., 2009). MI is closely linked to moral distress, as both arise from exposure to morally challenging situations but differ in their psychological consequences (Grimell and Nilsson, 2020). Whereas moral distress may be a temporary experience, MI represents a more enduring burden that can emerge when moral distress accumulates or intensifies (Linzer and Poplau, 2021). MI is characterized by the perception of having transgressed one’s moral code, whether through acts of commission, omission, or witnessing morally troubling events, often resulting in intense feelings of guilt, shame, self-condemnation, spiritual conflict, and difficulties making sense of the experience (Frankfurt and Frazier, 2016). It may also involve experiences of betrayal by trusted individuals or institutions in positions of legitimate authority (Shay, 2010). A growing body of research has linked MI to a range of adverse outcomes, including alcohol misuse (Battles et al., 2019; Grimell, 2023), social withdrawal (Hall et al., 2022), posttraumatic stress symptoms, depression, and burnout—a related but conceptually distinct construct that, unlike MI, does not necessarily involve moral transgressions (Williamson et al., 2018; Linzer and Poplau, 2021; Coimbra et al., 2024).

Although the concept of MI was originally developed in military populations, increasing attention has been given to MI among healthcare professionals (HCPs, ter Heide and Olff, 2023). Clinical practice frequently requires decisions involving competing ethical obligations, uncertainty, and the possibility of adverse outcomes (Čartolovni et al., 2021). These challenges became particularly salient during the COVID-19 pandemic, when healthcare systems operated under unprecedented strain (Rosen et al., 2022). HCPs were confronted with difficult decisions regarding the allocation of scarce resources, restrictions on family visitation, and the provision of care under rapidly changing clinical and organizational conditions (D’Alessandro et al., 2022). At the same time, they often faced institutional pressures, staffing shortages, overwhelming workloads, and policies that conflicted with their professional values or standards of care (French et al., 2022). Such circumstances may increase exposure to potentially morally injurious events and contribute to lasting psychological distress (Coimbra et al., 2024).

Within the European context, Sweden represented a distinctive case during the COVID-19 pandemic (Björkman et al., 2023). In line with a high level of public trust, albeit in contrast to many neighboring countries, Swedish authorities relied primarily on voluntary recommendations and individual responsibility rather than extensive mandatory restrictions (Kavaliunas et al., 2020; Ludvigsson, 2023). This approach sought to balance infection control with the preservation of personal freedoms and societal functioning (Ludvigsson, 2023). However, compared with other European countries, Sweden experienced substantially higher COVID-19 mortality during the early phases of the pandemic, generating considerable debate regarding its public health strategy (Kamerlin and Kasson, 2020). Consequently, Swedish HCPs operated within a healthcare and policy environment that differed from that of many other European countries (Claeson and Hanson, 2021). Such a context may have shaped HCPs’ experiences of moral and ethical challenges in ways that differ from those reported in other settings (Gustavsson et al., 2023). However, despite Sweden’s distinctive pandemic context and the growing literature on MI among HCPs, no psychometric validation studies of MI instruments have been yet conducted in Sweden.

The Moral Injury Symptoms Scale–Health Professional (MISS-HP) is a widely used instrument for assessing MI symptoms among HCPs. The original 10-item version, developed in the US, demonstrated satisfactory psychometric properties and a three-factor structure comprising Guilt/Shame, Spiritual Struggles, and Condemnation (Mantri et al., 2020). Since then, the MISS-HP has been translated and psychometrically evaluated in several countries, including China (Zhizhong et al., 2020), Czechia (Vlckova et al., 2025), Germany (Trifunovic-Koenig et al., 2022), Iran (Malakoutikhah et al., 2022), Turkey (Üstün, 2022), and Peru (Cabanillas-Chavez et al., 2024). However, findings across these studies have been inconsistent, with differences reported in the number of factors, retained items, and factor composition. Such variability may reflect cultural differences, the complexity of MI as a construct, or inconsistencies in the performance of specific MISS-HP items across settings (Cabanillas-Chavez et al., 2024). Given the absence of psychometric studies of MI instruments in Sweden and the distinctive context in which the COVID-19 pandemic unfolded in the country, the present study aimed to translate and evaluate the psychometric properties of the Swedish MISS-HP in a sample of hospital-based HCPs from across Sweden and determine whether the instrument provides a reliable tool for future research on MI in Swedish healthcare settings.

## 2 Methods

### 2.1 Procedures

We used an online survey to recruit a convenience sample of HCPs across Sweden between January 14th and May 29th, 2026. The survey link was distributed to all 14 regions in Sweden with a request for further dissemination to the hospitals within the region, and subsequently to all HCPs working primarily in advanced or acute care settings. The link was also distributed with aid of national professional associations for physicians and nurses. Data were collected anonymously using Research Electronic Data Capture (REDCap). No identifying information, including workplace identifiers, was collected. Only participants who completed the entire survey were included in the analyses to ensure adequate response quality. The electronic survey was configured to retain data only upon completion; therefore, partial responses were not recorded. Participation was voluntary, and no financial or other incentives were provided. Eligible participants were HCPs actively involved in patient care in Swedish hospitals who provided informed consent prior to participation. Ethical approval was obtained from the Swedish Ethical Review Authority through Umeå University (Dnr: 2025–06740-01).

### 2.2 Swedish MISS-HP

The MISS-HP is a self-report instrument designed to assess MI symptoms among HCPs. The original version contains 10 items rated on a 10-point Likert scale, yielding total scores ranging from 10 to 100, with higher scores indicating greater MI symptom severity (Mantri et al., 2020). The items cover key dimensions of MI, including guilt, shame, and betrayal, loss of trust, self-condemnation, and spiritual struggles. Four items (MISS-5, MISS-6, MISS-7, and MISS-10) are reverse scored. In addition, a separate item evaluates the extent to which MI symptoms interfere with daily functioning. The original study suggested a cut-off score of 36 or higher as indicative of clinically significant MI symptoms (Mantri et al., 2020).

The Swedish version was initially translated by a bilingual nurse researcher (native Swedish speaker). The translation was subsequently reviewed by a group of Swedish researchers (both native English and native Swedish speakers) with expertise in survey data, MI and healthcare ethics. Any discrepancies or concerns regarding wording were discussed and resolved through consensus. In addition, all survey respondents were able to provide feedback on the survey. No feedback was provided regarding the comprehensibility of the instrument’s wording.

### 2.3 Statistical analyses

Descriptive statistics were used to summarize participants’ sociodemographic characteristics, including age, gender, religious affiliation, marital status, profession, and workplace setting. As an initial step, parallel analysis was conducted to estimate the number of factors underlying the Swedish MISS-HP. Subsequently, exploratory factor analysis (EFA) was performed using maximum likelihood extraction and oblimin rotation. Factor solutions were iteratively refined by examining item loadings, model fit, and interpretability (Fabrigar et al., 1999). Items with factor loadings below 0.40 were removed because they did not load adequately on any latent factor, and revised models were re-estimated until a satisfactory and interpretable solution was obtained (Howard, 2016). Model fit was evaluated using the Tucker–Lewis Index (TLI), Root Mean Square Error of Approximation (RMSEA), Standardized Root Mean Square Residual (RMSR), Bayesian Information Criterion (BIC), and the chi-square test. TLI values ≥ 0.90, RMSEA and RMSR values ≤ 0.08, and a non-significant chi-square test were considered indicative of acceptable model fit (Hu and Bentler, 1999).

To identify a candidate cut-off score for the Swedish MISS-HP, receiver operating characteristic (ROC) analysis was performed. Consistent with the original MISS-HP study (Mantri et al., 2020), functional impairment was assessed using item 11, which measures the extent to which MI symptoms interfere with daily functioning. Responses were dichotomized into low impairment (“not at all” or “mild”) and moderate-to-severe impairment (“moderate,” “very much,” or “extremely”). The candidate cut-off score was determined using Youden’s index. The area under the curve (AUC) was used to evaluate the scale’s ability to discriminate between participants with and without clinically relevant impairment, with values of 0.80 or higher indicating good discriminative performance (Çorbacıoğlu and Aksel, 2023).

Internal consistency was assessed using McDonald’s Omega, inter-item correlations, and corrected item–total correlations. Evidence for construct validity was examined using bivariate Spearman’s rank-order correlations between MISS-HP scores and measures of psychological distress. Spearman’s correlations were used because all study variables deviated significantly from normality according to the Shapiro–Wilk test (all *p* values < 0.001). The following instruments were included: (1) the Patient Health Questionnaire-9 (PHQ-9), a nine-item measure assessing depressive symptoms using a four-point response scale (Kroenke et al., 2001); (2) the Generalized Anxiety Disorder-7 (GAD-7), a seven-item measure assessing anxiety symptoms using a four-point response scale (Spitzer et al., 2006); and (3) the Professional Quality of Life Scale (ProQOL), a 30-item measure comprising the subscales Compassion Satisfaction, Burnout, and Secondary Traumatic Stress. The ProQOL was retrieved from the official website^1^. In the present study, internal consistency coefficients were *α* = 0.90 for the PHQ-9, *α* = 0.90 for the GAD-7, *α* = 0.91 for Compassion Satisfaction, *α* = 0.83 for Burnout, and *α* = 0.77 for Secondary Traumatic Stress. Based on previous research (Coimbra et al., 2024), we expected positive associations between MISS-HP scores and depression, anxiety, burnout, and secondary traumatic stress, as well as negative associations with compassion satisfaction. All statistical analyses were performed using packages *psych* (Revelle and Revelle, 2015) and *pROC* (Robin et al., 2011) in R version 4.5.2.

## 3 Results

### 3.1 Demographics

We included 585 HCPs who completed all items of the MISS-HP. The sample was predominantly women (*n* = 425, 72.2%), married or in a civil partnership (*n* = 468, 80.7%), and had no religious affiliation (*n* = 302, 51.4%). The majority of participants were nurses (*n* = 303, 51.4%), followed by assistant nurses (*n* = 134, 22.8%) and physicians (*n* = 128, 21.7%). Participants most commonly worked in intensive care units (*n* = 186, 31.6%) and surgery units (*n* = 174, 29.5%), followed by inpatient hospital wards (*n* = 135, 22.9%), emergency rooms (*n* = 101, 17.1%), and ambulance care services (*n* = 47, 8.0%). Participants had a mean age of 45.95 years (SD = 10.84).

### 3.2 Exploratory factor analysis

Parallel analysis suggested a three-factor solution for the Swedish MISS-HP. Accordingly, an exploratory factor analysis (EFA) specifying three factors was conducted to examine the underlying structure of the scale. MISS-7 did not load on any factor, whereas MISS-10 loaded negatively on a third factor and was therefore considered conceptually problematic. Both items were removed, and the EFA was re-estimated. However, the third factor remained poorly defined, being represented by a single substantive item (MISS-9). Because a latent factor represented by only one substantive item cannot be considered a stable psychometric dimension, the three-factor solution was considered inadequate rather than theoretically unsupported. Consequently, a more parsimonious two-factor solution was examined.

A two-factor EFA was subsequently conducted using all ten MISS-HP items. MISS-1, MISS-7, MISS-9, and MISS-10 failed to load on either factor (*λ* < 0.40) and were therefore removed. The EFA was then re-estimated using the remaining six items. The resulting two-factor solution demonstrated good fit to the data [*χ*^2^(4) = 9.07, *p* = 0.059; RMSEA = 0.046; TLI = 0.975; RMSR = 0.018] and accounted for 43.6% of the total variance. The first factor, labeled Guilt/Shame, comprised four items (MISS-2, MISS-3, MISS-4, and MISS-8) and explained 31.7% of the variance, whereas the second factor, labeled Trust/Meaning, comprised two items (MISS-5 and MISS-6) and explained 11.9% of the variance. Factor loadings ranged from 0.47 to 0.82, and the correlation between the two factors was moderate (*r* = 0.34). The mean score of the six-item scale was 16.07 (SD = 8.24) and the possible range score is between 6 and 60. The final factor loadings and retained items of the Swedish MISS-HP are presented in Table 1.

**Table 1:** Factor loadings for the final two-factor exploratory factor analysis of the six-item Swedish MISS-HP (*n* = 585).

Factor loading		
Swedish MISS-HP items	Factor 1 (guilt/shame)	Factor 2 (trust/Meaning)
2. Jag känner skuld över att jag inte lyckats rädda någon från att bli allvarligt skadad eller dö (*I feel guilt over failing to save someone from being seriously injured or dying)*	0.674	
3. Jag skäms över vad jag har gjort eller inte gjort när jag har vårdat mina patienter *(I feel ashamed about what I’ve done or not done when providing care to my patients)*	0.821	
4. Jag besväras av. att ha handlat på ett sätt som överträtt min egen moral eller mina egna värderingar *(I am troubled by having acted in ways that violated my own morals or values)*	0.738	
5. De flesta människor som jag arbetar med i egenskap av. vårdpersonal är pålitliga (*Most people with whom I work as a health professional are trustworthy)*		0.660
6. Jag har en god förståelse för vad som gör mitt liv som vårdpersonal meningsfullt (*I have a good sense of what makes my life meaningful as a health professional)*		0.491
8. På det hela taget är jag benägen att känna att jag är ett misslyckande i mitt arbete som vårdpersonal *(All in all, I am inclined to feel that I’m a failure in my work as a health professional)*	0.465	

Factor loadings ≥ 0.40 are considered salient. Excluded items were MISS-1: Jag känner mig sviken av annan vårdpersonal som jag tidigare litade på (I feel betrayed by other health professionals whom I once trusted); MISS-7: Jag har förlåtit mig själv för vad som har hänt mig eller andra som jag har vårdat (I have forgiven myself for what’s happened to me or to others whom I have cared for); MISS-9: Ibland upplever jag att Gud straffar mig för vad jag har gjort eller inte gjort när jag har vårdat patienter (I sometimes feel God is punishing me for what I’ve done or not done while caring for patients); MISS-10: Jämfört med innan jag gick igenom dessa upplevelser har min religiösa/andliga tro stärkts (Compared to before I went through these experiences, my religious/spiritual faith has strengthened).

We also examined whether a one-factor solution would provide a more parsimonious representation of the six-item Swedish MISS-HP. In this model, MISS-5 and MISS-6 showed poor factor loadings and the overall fit was unsatisfactory [*χ*^2^(9) = 75.68, *p* < 0.001; RMSEA = 0.112; TLI = 0.856; RMSR = 0.080]. Moreover, the one-factor solution yielded a substantially higher BIC than the two-factor solution (ΔBIC = 34.72). Therefore, the two-factor six-item version demonstrated superior fit and was retained as the final Swedish MISS-HP.

### 3.3 Reliability and construct validity

Omega total for the final six-item Swedish MISS-HP was 0.80, indicating good internal consistency. Inter-item correlations ranged from 0.12 to 0.61, and corrected item–total correlations ranged from 0.26 to 0.61. Construct validity was examined through associations with depression, anxiety, burnout, secondary traumatic stress, and compassion satisfaction (Table 2). Correlations were in the expected directions, with higher MISS-HP scores associated with greater psychological distress and lower compassion satisfaction.

**Table 2:** Construct validity (six-item Swedish MISS-HP total score and subscale scores).

Measures	MISS-HP total	Guilt/Shame subscale	Trust/Meaning subscale
Construct validity			
Depression (PHQ-9)	0.46	0.42	0.33
Anxiety (GAD-7)	0.48	0.45	0.33
Burnout (ProQOL)	0.52	0.43	0.42
Secondary traumatic stress (ProQOL)	0.45	0.45	0.27
Compassion satisfaction (ProQOL)	−0.42	−0.31	−0.39

All values are Spearman’s correlations and significant at the <0.001 level.

### 3.4 Candidate cut-off determination

**Figure 1:**
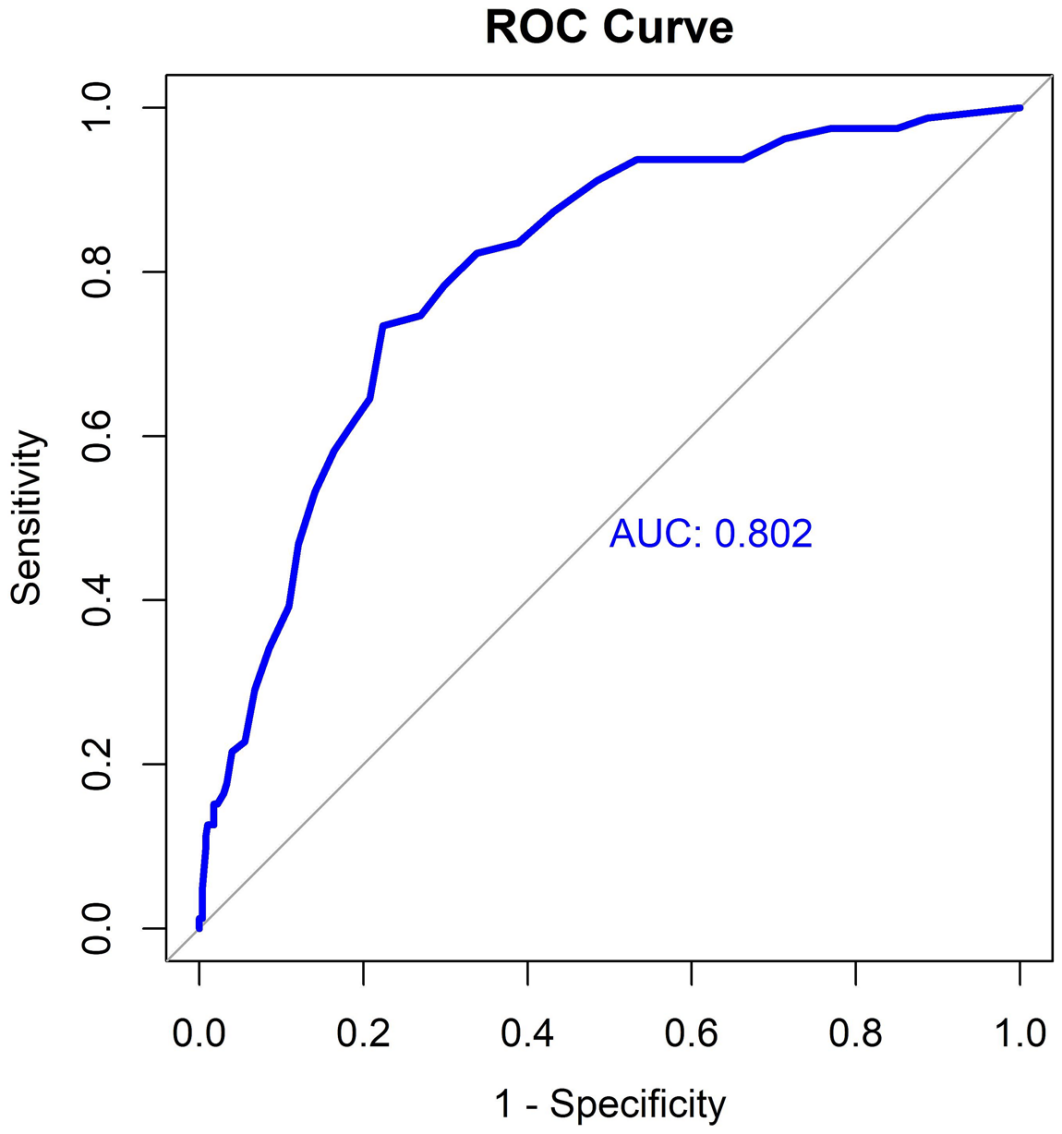
Receiver operating characteristic (ROC) curve for the six-item Swedish MISS-HP.

The receiver operating characteristic (ROC) curve is presented in Figure 1. The area under the curve (AUC) was 0.802, indicating good discriminative performance. Using Youden’s index, the optimal cut-off score for the six-item Swedish MISS-HP was identified as 20 or higher. At this threshold (≥ 20), sensitivity was 73.4% and specificity was 77.6% for distinguishing HCPs reporting at least moderate impairment associated with MI symptoms. In the present sample, 171 participants (29.2%) scored at or above the proposed cut-off.

## 4 Discussion

In the present study, we examined the psychometric properties of the Swedish version of the MISS-HP in a nationwide sample of hospital-based HCPs. The findings supported a two-factor solution comprising six of the original ten items, with factors reflecting Guilt/Shame and Trust/Meaning. The final version demonstrated satisfactory internal consistency, good model fit, and good discriminative ability. Furthermore, MISS-HP scores were associated with measures of psychological distress and compassion satisfaction in the expected directions, providing evidence of construct validity. Taken together, these findings suggest that the six-item Swedish MISS-HP demonstrated satisfactory psychometric properties in the present sample and may be useful for future research in Swedish hospital settings.

Consistent with previous validation studies, the factor structure identified in the present study differed from that proposed in the original MISS-HP, adding to evidence of substantial cross-cultural variability in the instrument’s structure (Zhizhong et al., 2020; Vlckova et al., 2025). Notably, these differences may reflect not only cultural influences but also methodological differences across validation studies, including variations in sample characteristics and analytical approaches. In the Swedish sample, both religion-related items (MISS-9 and MISS-10) failed to load adequately on any factor and were therefore removed from the final factor solution. One possible explanation is the relatively secular nature of Swedish society. In our sample, more than half of the participants reported having no religious affiliation, suggesting that religion-based items may be less applicable or meaningful for a substantial proportion of respondents. For example, MISS-9 *(“I sometimes feel God is punishing me for what I’ve done or not done while caring for patients”)* presupposes belief in God and may therefore be less relevant for some respondents. In addition, MISS-10 *(“Compared to before I went through these experiences, my religious/spiritual faith has strengthened”)* may be open to multiple interpretations (Trifunovic-Koenig et al., 2022). It is possible that individuals experiencing MI may report stronger faith as a coping response, while others may endorse the item for reasons unrelated to MI, such as stable or increasing religious commitment over time. Interestingly, this item also failed to load adequately in the Persian (Malakoutikhah et al., 2022), Peruvian (Cabanillas-Chavez et al., 2024), and German (Trifunovic-Koenig et al., 2022) validation studies, suggesting that its psychometric instability may not be unique to the Swedish context. Together, these findings highlight the importance of carefully evaluating the cross-cultural relevance of religion-related items when adapting instruments designed to assess MI.

Additional discrepancies were observed for MISS-1 and MISS-7, which also failed to load adequately in the Swedish sample. Interestingly, MISS-1 *(“I feel betrayed by other health professionals whom I once trusted”*) has likewise shown poor psychometric performance in the Peruvian and Persian validation studies (Malakoutikhah et al., 2022; Cabanillas-Chavez et al., 2024). One possible explanation is that the item may not fully reflect the betrayal dimension originally described in the MI literature. Classic conceptualizations of MI emphasize betrayal by legitimate authorities in high-stakes situations (Shay, 2010), whereas MISS-1 focuses on betrayal by other healthcare professionals. MISS-7 (*“I have forgiven myself for what’s happened to me or to others whom I have cared for”)* also demonstrated poor psychometric performance in the Peruvian validation study. An explanation proposed by the authors was that the item may be semantically ambiguous because it simultaneously refers to forgiving oneself for what happened to oneself and for what happened to patients under one’s care (Cabanillas-Chavez et al., 2024). Overall, these findings suggest that some MISS-HP items may require further conceptual and psychometric refinement to improve their cross-cultural applicability. Accordingly, the Swedish MISS-HP should be viewed as a culturally adapted measure of MI rather than a direct structural replication of the original instrument, as it remains unclear whether the excluded domains reflect genuine cross-cultural differences or the limited psychometric performance of specific items.

In our study, the Guilt/Shame factor was composed of MISS-2, MISS-3, MISS-4, and MISS-8. Notably, MISS-2, MISS-3, and MISS-4 demonstrated strong factor loadings and have consistently clustered together across the original and most translated versions of the MISS-HP (Mantri et al., 2020; Zhizhong et al., 2020; Malakoutikhah et al., 2022). This pattern suggests that guilt and shame-related experiences may represent one of the most stable and cross-culturally replicable dimensions of MI among HCPs. In the Swedish sample, MISS-8 *(“All in all, I am inclined to feel that I’m a failure in my work as a health professional”)* also loaded on this factor. Although originally conceptualized as part of the condemnation dimension, feelings of professional failure may be closely linked to self-blame, guilt, and negative self-evaluation following perceived moral transgressions. The inclusion of MISS-8 within the Guilt/Shame factor therefore appears conceptually coherent and may reflect the tendency for self-condemnation and guilt-related cognitions to overlap in the experience of MI.

Furthermore, the second factor (Trust/Meaning) comprised MISS-5 *(“Most people with whom I work as a health professional are trustworthy”)* and MISS-6 *(“I have a good sense of what makes my life meaningful as a health professional”)*. Although represented by only two items, this factor is not entirely inconsistent with previous validation studies. Both items clustered together in the original MISS-HP and in several translated versions (Mantri et al., 2020; Zhizhong et al., 2020; Üstün, 2022), albeit alongside additional items. Conceptually, both items reflect positive beliefs that may be challenged following potentially morally injurious experiences, namely trust in others and a sense of professional meaning and purpose. The Trust/Meaning factor identified in the Swedish sample may therefore capture an important dimension of MI related to disruptions in interpersonal trust and professional meaning.

This study has several limitations. First, the use of a convenience sample may have introduced selection bias and limits the generalizability of the findings. Second, although the Swedish translation was reviewed by bilingual experts, a formal cross-cultural adaptation procedure involving independent back-translation was not performed. Although consensus among bilingual experts was used to ensure conceptual equivalence, the absence of back-translation may have contributed to the instability of some items and should be considered when interpreting the present findings. Third, the final two-factor solution included a factor represented by only two items (Trust/Meaning), which may have implications for the stability of this dimension. However, two-item factors have also been identified in previous MISS-HP validation studies (Malakoutikhah et al., 2022; Cabanillas-Chavez et al., 2024; Vlckova et al., 2025), suggesting that this finding is not unique to the Swedish version. Fourth, the candidate cut-off score was derived using a self-reported indicator of functional impairment. Its performance should be further examined and validated using external clinical criteria. Finally, given the substantial variability in factor structures reported across previous MISS-HP validation studies, the present study adopted an exploratory rather than confirmatory approach. Future studies should seek to replicate the proposed factor structure in independent samples and further evaluate its stability using confirmatory factor analysis. They should also examine the temporal stability of the instrument, measurement invariance across different healthcare professional groups and gender, and its convergent validity with related constructs.

However, the study also has important strengths. It is the first psychometric evaluation of a MI instrument in Sweden and was conducted in a nationwide sample of hospital-based HCPs. Moreover, despite the removal of four items, the final six-item version demonstrated satisfactory psychometric properties across multiple indicators. Given the distinctive context in which the COVID-19 pandemic unfolded in Sweden, a Swedish measure with preliminary evidence of validity and reliability may facilitate future research on MI among Swedish HCPs.

In conclusion, the present findings suggest that the Swedish MISS-HP is best represented by a concise two-factor structure comprising Guilt/Shame and Trust/Meaning. The resulting six-item Swedish MISS-HP demonstrated satisfactory psychometric properties in the present sample, providing supporting evidence for validity and reliability. These findings support its potential use as a measure of MI symptoms in Swedish hospital settings and may facilitate future research on MI among Swedish HCPs.

## Data Availability

The datasets presented in this article are not readily available because data sharing is regulated in legal agreements. Requests to access the datasets should be directed to AH, anna.hjalmarsson@umu.se.

## Footnotes

1
http://www.proqol.org
